# Validation of Metabolic Alterations in Microscale Cell Culture Lysates Using Hydrophilic Interaction Liquid Chromatography (HILIC)-Tandem Mass Spectrometry-Based Metabolomics

**DOI:** 10.1371/journal.pone.0154416

**Published:** 2016-04-27

**Authors:** Venugopal Gunda, Fang Yu, Pankaj K. Singh

**Affiliations:** 1 Eppley Institute for Research in Cancer and Allied Diseases, University of Nebraska Medical Center, Omaha, Nebraska, United States of America; 2 Department of Biostatistics, University of Nebraska Medical Center, Omaha, Nebraska, United States of America; 3 Department of Pathology and Microbiology, University of Nebraska Medical Center, Omaha, Nebraska, United States of America; 4 Department of Biochemistry and Molecular Biology, University of Nebraska Medical Center, Omaha, Nebraska, United States of America; 5 Department of Genetics, Cell Biology and Anatomy, University of Nebraska Medical Center, Omaha, Nebraska, United States of America; Roswell Park Cancer Institute, UNITED STATES

## Abstract

By standard convention, in order to increase the efficacy of metabolite detection from cell culture lysates, metabolite extracts from a large quantity of cells are utilized for multiple reaction monitoring-based metabolomic studies. Metabolomics from a small number of cell extracts offers a potential economical alternative to increased cell numbers, in turn increasing the utility of cell culture-based metabolomics. However, the effect of reduced cell numbers on targeted metabolomic profiling is relatively unstudied. Considering the limited knowledge available of the feasibility and accuracy of microscale cell culture metabolomics, the present study analyzes differences in metabolomic profiles of different cell numbers of three pancreatic cancer cell lines. Specifically, it examines the effects of reduced cell numbers on metabolite profiles by obtaining extracts either directly from microscale culture plates or through serial dilution of increased numbers of cellular metabolite extracts. Our results indicate reduced cell numbers only modestly affect the number of metabolites detected (93% of metabolites detected in cell numbers as low as 10^4^ cells and 97% for 10^5^ cells), independent of the method used to obtain the cells. However, metabolite peak intensities were differentially affected by the reduced cell numbers, with some peak intensities inversely proportional to the cell numbers. To help eliminate such potential inverse relationships, peak intensities for increased cell numbers were excluded from the comparative analysis. Overall, metabolite profiles from microscale culture plates were observed to differ from the serial dilution samples, which may be attributable to the medium-to-cell-number ratios. Finally, findings identify perturbations in metabolomic profiling for cellular extracts from reduced cell numbers, which offer future applications in microscale metabolomic evaluations.

## Introduction

Metabolomics analyses enable profiling of metabolites from biological samples of both endogenous and exogenous origin [1]. Metabolites extracted from biological samples are analyzed either using an untargeted metabolomic approach to qualitatively identify metabolites or using a targeted metabolomic approach to quantitatively measure the absolute levels of metabolites [2]. The targeted metabolomics approach is based on a strategy to detect metabolites that utilizes the characteristic and unique mass/charge (m/z) values for individual metabolites. This targeted methodology has the advantage over untargeted/qualitative metabolomics in that it enhances the authenticity of metabolite identification and quantitation [3]. Both multiple reaction monitoring (MRM) and selected reaction monitoring (SRM) methodologies applied to targeted metabolomics use the unique m/z values to detect multiple metabolites present in biological sample in a single, targeted metabolomics platform [4,5]. Applying the unique m/z values facilitates relative quantitation of metabolites from matrices of similar biological origins, which has wide applicability in identifying metabolic perturbations that occur in both physiological and pharmacological interventions [6].

Successful relative quantitation of metabolites using MRM methodology, however, depends on the abundance and resolution of metabolites present within biological samples of interest. The abundance of metabolites is, in turn, expected to be affected by several factors, such as the nature and quantity of the biological sample used for metabolite extraction, as well as the method for sample collection, analyses of metabolites, and preservation [7]. Most sample induced-factors that affect the abundance of metabolites are controlled by utilizing optimized methods of sample preparation and extraction applied to biological samples maintained under control conditions, such as cell cultures.

*In vitro* cell culture provide an optimal matrix for metabolomic analysis applicable in identifying metabolic perturbations under controlled conditions [8]. Metabolite extracts obtained from *in vitro* cultured cells constitute a multitude of pathways, with a wide range of concentrations affected by the cell growth phase and treatments the cultured cells undergo [9,10]. Therefore, taking these factors into consideration, in order to obtain concentrated samples and reduce loss of metabolite abundance, metabolite extracts obtained from highly dense cell cultures are generally utilized for analysis in metabolomics [11,12]. Still, there has been little emphasis on the effects that reduced cell numbers have on metabolomics [13], leaving the potential of microscale metabolomics understudied.

Recent developments in cellular metabolomics have focused on microscale culture-based metabolomics, wherein cells cultured in microscale culture dishes, such as 96-well plates, are utilized for metabolomic analyses. Considering the economic factors associated with highly dense cell cultures, microscale approaches have the potential to increase the utility and feasibility of microscale cultures in metabolomics [14]. In addition to the reduced cost of microscale cultures, they appear to represent optimal models for cytotoxicity evaluations in high-throughput screening assays [15]. Another rationale supporting microscale metabolomics approach, requiring a reduced cell number, is that it can identify metabolomic perturbations, as shown for single yeast cells [16]. Even less evaluated, however, are limitations of microscale culture methods on metabolomic profiling, with few findings that indicate whether diluted samples reflect the metabolome of microscale cultured cell lines [13].

The study herein applied a targeted metabolomic approach to assess the effects of reduced cell numbers on metabolomic profiling using cell lysates obtained from reduced cell numbers in culture. Relative quantitation-targeted metabolomic analyses were performed to compare the effects that sample dilution and direct extraction of metabolites from microscale cultures have on the abundance of metabolites. Results demonstrated that metabolite peak intensities, rather than the total number of metabolites detected, are most affected by the reduced cell numbers. The number and profile of metabolites detected, on the other hand, remain rather independent of either dense or sparse cell cultures.

## Materials and Methods

### Cell Culture and Cell Number Evaluations

Pancreatic cancer cell lines S2-013, CFPAC, and HUPT3 were obtained from American Type Culture Collection (ATCC) and cultured in Dulbecco’s Modified Eagle Medium (DMEM) with 10% fetal bovine serum (FBS) in 6 cm culture dishes and 24-, 12- and 96-well plates. Medium exchange occurred for 12 hours before metabolite extraction; cells were maintained in culture under normal conditions. Three replicates of in each well-plate or cell culture dishes were seeded for metabolite extraction, and an additional replicate was used for cell counting, which was analyzed with the trypan blue exclusion method. Briefly, cells were rinsed with phosphate buffered saline (PBS), trypsinized for 2 minutes, centrifuged, and counted using a BioRad cell counter and the trypan blue exclusion method. Final cell counts were adjusted for the dilution factor.

### Polar Metabolite Extraction

Polar metabolites were extracted from cells cultured in different culture dishes following our previously published method, with necessary modifications discussed herein [17]. Briefly, media was removed from the culture dishes, and cells were rinsed quickly with liquid chromatography-mass spectrometry (LC-MS)-grade water. To avoid cell lysis during aqueous rinsing, a multichannel pipet was used to add and immediately remove 100 μl of LC-MS-grade water from each well of a 96-well plate, followed by the addition of 100 μl of cold, 80% methanol and ^13^C-labeled glutamine added as an internal standard. Next, the 96-well plates were placed on dry ice and transferred to -80°C for 10 minutes. Post incubation, methanol lysates were collected in individual polypropylene tubes and processed as described previously [17]. A similar method was applied for the polar metabolite extraction from other culture dishes, but with a proportionate increase in the volume of 80% methanol used for the metabolite extraction.

### Sample Preparation and Dilutions

Lyophilized cell lysates prepared from 6 cm, and 24- and 96-well culture plates were stored at -80°C before being dissolved in LC-MS grade water. Samples obtained from the different sized culture dishes were dissolved in proportionate volumes of LC-MS grade water based on the cell counts (10 μl water for approximately 2 x 10^6^ cells from the 6 cm culture dishes, 10 μl for each 2 x 10^5^ cells from the 24-well culture plates, and 10 μl for 2 x 10^4^ cells from 96-well culture plates) and centrifuged at 13,000 rpm, 4°C for 5 minutes. Supernatants were transferred into glass vials with conical inserts, and equal volumes of samples (5 μl) were injected through autosampler for LC-MS/MS analyses. Samples from the 6 cm dishes were further diluted to 2x, 10x, and 100x using LC-MS grade water. Next, 5 μl of the diluted samples were injected into the autosampler for LC-MS/MS analysis.

### Liquid Chromatography and Tandem Mass Spectrometry

Hydrophilic interaction liquid chromatography (HILIC) and tandem mass spectrometry (MS/MS), with highly selective positive and negative modes of electron spray ionization (ESI), were applied to the relative quantitative analysis of metabolic intermediates encompassing diverse pathways, including glycolysis, the citrate cycle, the pentose phosphate pathway, the urea cycle, as well as metabolism of nucleotides, amino acids, vitamins, and a few lipids, as described previously [4].

### Metabolomic Analysis

Reproducibility of our LC method was confirmed based on consistencies in the retention time of peaks corresponding to ^13^C-glutamine, which was added as internal standard. Authenticity of the MRM method was confirmed by comparing the m/z patterns of commercially available standards with published m/z patterns. MRM data was acquired by utilizing Analyst® software (SCIEX Inc.), and peak integration was performed with Multiquant® (SCIEX Inc.), Integrated peak areas were applied to relative quantitation analysis.

### Data Analysis

Metabolite peaks with signal-to-noise (S/N) ratios ≥ 10^4^ recorded among the three technical or biological replicates were considered in the relative quantitation analysis. Metabolites that showed consistent peak areas among the three replicates (p-value < 0.05) were considered to evaluate the total number of metabolites detected in each sample and for its subsequent relative analysis. Mean and standard error of mean (SEM) were obtained for peak areas of each metabolite from three technical or biological replicate samples using Microsoft™ Excel. These values were log transformed and applied to non-linear regression analysis to obtain the slope and coefficient of regression (R^2^), which were in turn processed through Graphpad™ Prism 5 to evaluate the linearity of metabolite abundance corresponding to the sample dilution or cell number. Venn diagrams were created by using the online source Venny 2.0.2 (bioinfogp.cnb.csic.es/). Unsupervised hierarchical clustering analyses and partial least squares-discriminant analyses (PLS-DA) were performed using Metaboanalyst 2.0 (www.metaboanalyst.ca).

## Results and Discussion

### Effect of Reduced Cell Numbers on Metabolite Detection

Mass spectrometry (MS) coupled with chromatography is a highly sensitive and reproducible analytical platform to improve separation, resolution, and detection of multiple analytes from complex mixtures, including biological matrices [18,19]. Owing to its increased sensitivity and resolution, hydrophilic interaction liquid chromatography-based tandem mass spectrometry (HILIC-MS/MS) is particularly applicable to relative quantitative analyses of multiple polar metabolites obtained from cell lysates [11]. Even with the high sensitivity achievable using HILIC-MS/MS, metabolite detection still depends on the abundance of different metabolites in the biological samples used for metabolomic analysis. In general, optimal metabolite abundance from cultured cell lysates is thought to be achieved by obtaining metabolite extracts from an increased number of cultured cells [11]. Due to the costs associated with *in vitro* cell cultures, metabolomic analysis from cultures with reduced cell numbers are being considered for increasing the utility of *in vitro* metabolomic analyses [8,13]. However, potential changes in metabolomic patterns for decreased cell numbers obtained from microscale culture dishes, such as 96-well plates, on are under-studied, even despite the wide applications of microscale cultures in pharmacological and toxicological evaluations [20].

To help fill this gap in knowledge, this study evaluated if decreasing cell numbers affected the total number of metabolites detectable using a targeted metabolomics approach. Our study applied two different methodologies to obtain metabolites from a reduced number of cells in culture from the pancreatic cancer cell line S2-013. As discussed in the methods section, one method consisted of seeding pre-quantified cell numbers in 6 cm dishes, and 24- and 96-well culture plates for 12 hours and extracting metabolites directly from these cultured cells. In the second approach, cell lysates obtained from cells cultured in 6 cm dishes (approximately 2x10^6^ cells) were diluted 10x and 100x to obtain lysates equivalent to those of low cell numbers obtained from 24- and 96-well culture plates, respectively. The final volume of the sample extracts injected for MS analyses were equivalent to 10^6^ cells for 6 cm dish cultures, or a 1x dilution, 10^5^ cells for 24-well plate cultures, or a 10x dilution, and 10^4^ cells for 96-well plate cultures, or a 100x dilution. Using the LC-MS/MS-based MRM approach, the maximum number of metabolites (i.e., 190) was detected for the S2-013 cell lysates from 10^6^ cells (6 cm dish cultures), followed by extracts from 10^5^ cells (the 24-well plate) (Table 1, metabolites listed in Table 2). The total number of polar metabolites decreased modestly corresponding to a decrease in the cell number, with the fewest metabolites detected in the 100x dilution (of 10^6^ cells) samples (i.e., 163), as summarized in Table 1. Thus, our analysis indicates that the number of detectable metabolites is modestly altered by changes in cell numbers, which is in agreement with another metabolomic study wherein metabolite abundance was only slightly affected by the serial dilution of metabolite extracts [13]. Furthermore, our evaluation of trends in the total number of metabolites detected from cell lysates, obtained through two different methodologies, indicated that the method to obtain reduced cell numbers only modestly affects the total number of metabolites detected using a common LC-MS/MS approach.

**Table 1:** Summary of total number of metabolites detected with different cell numbers.

Sample source	Cell number per sample injections	Number of metabolites detected
6 cm dish cultures	10^6^	190
24 well plate cultures	10^5^	187
96 well plate cultures	10^4^	169
10x dilution of 10^6^ cell lysate	10^5^	177
100x dilution of 10^6^ cell lysate	10^4^	163

**Table 2:** Summary of metabolites included for metabolomic analysis.

Metabolite	Identifier	Metabolite	Identifier
Isobutyrylglycine	CHEBI:70979	S-Adenosyl-L-methionine	CHEBI:15414
Serine	CHEBI:17115	S-Adenosyl-L-methioninamine	CHEBI:67040
3-phospho-serine	CHEBI:37712	Glutathione	CHEBI:16856
Phosphoserine	CHEBI:37712	Glutathione disulfide	CHEBI:58297
Betaine	CHEBI:17750	S-Ribosyl-L-homocysteine	CHEBI:17575
betaine aldehyde	CHEBI:15710	Creatine	CHEBI:16919
Dimethylglycine	CHEBI:17724	Creatinine	CHEBI:16737
Threonine	CHEBI:16857	Arginine	CHEBI:16467
Lysine	CHEBI:18019	1,4-Diaminobutane	CHEBI:17148
Pipecolic acid	CHEBI:17964	Ornithine	CHEBI:15729
Carnitine	CHEBI:17126	Citrulline	CHEBI:16349
Acetylcarnitine	CHEBI:73024	Allantoin	CHEBI:15676
L-alpha-Aminoadipate	CHEBI:37023	Urea	CHEBI:16199
Acetyllysine	CHEBI:17752	Sarcosine	CHEBI:15611
Alanine	CHEBI:16977	L-Arginino-succinate	CHEBI:15682
Asparagine	CHEBI:17196	N-Acetylputrescine	CHEBI:17768
Aspartate	CHEBI:29993	Carnosine	CHEBI:15727
Phenylalanine	CHEBI:17295	N-acetyl-L-ornithine	CHEBI:86496
Tyrosine	CHEBI:17895	Histidine	CHEBI:15971
Dopamine	CHEBI:18243	1-Methylhistamine	CHEBI:29009
Valine	CHEBI:16414	Imidazoleacetic acid	CHEBI:57969
2-hydroxyisobutyric acid	CHEBI:50129	1-Methyl-Histidine	CHEBI:70958
Leucine	CHEBI:15603	Proline	CHEBI:17203
Lipoamide	CHEBI:17460	Hydroxyproline	CHEBI:24741
methylsuccinic acid	CHEBI:30936	Glutamine	CHEBI:18050
Methionine	CHEBI:16643	Glutamate	CHEBI:29985
Methionine sulfoxide	CHEBI:17016	Phenylacetylglutamine	CHEBI:8087
Phosphorylcholine	CHEBI:18132	Tryptophan	CHEBI:16828
Choline	CHEBI:15354	Kynurenine	CHEBI:16946
Glycerophosphocholine	CHEBI:36313	4-Aminobutyrate	CHEBI:30566
dimethyl-L-arginine	CHEBI:17929	Indole	CHEBI:35581
Homocysteine	CHEBI:17588	β-Amino butyric acid	CHEBI:35621
Cystathionine	CHEBI:17482	Melatonin	CHEBI:16796
Cysteamine	CHEBI:17141	Metanephrine	CHEBI:6270
Methylcysteine	CHEBI:45658	γ-Aminoisobutyrate	CHEBI:30566
S-adenosyl-L-homoCysteine	CHEBI:16680	Tryptophanol	CHEBI:17890
3-hydroxy-anthranilate	CHEBI:36559	Mesaconic acid	CHEBI:16600
Hydroxy-tryptophan	CHEBI:84194	Mevalonic acid	CHEBI:25351
Glucosamine	CHEBI:5417	2-Aminooctanoic acid	CHEBI:75145
N-Acetyl-glucosamine	CHEBI:59640	L-α-Aminobutyrate	CHEBI:74359
Aminoimidazole carboxamide ribonucleotide	CHEBI:18406	Cobalamin	CHEBI:30411
Adenosine	CHEBI:16335	Pyridoxine	CHEBI:16709
1-Methyladenosine	CHEBI:16020	Pyridoxamine	CHEBI:16410
S-Methyl-5-thioadenosine	CHEBI:17509	Flavone	CHEBI:42491
Adenine	CHEBI:16708	Biotin	CHEBI:15956
AMP	CHEBI:16027	Thiamine phosphate	CHEBI:26945
dAMP	CHEBI:17713	Thiamine	CHEBI:18385
ADP	CHEBI:16761	Riboflavin	CHEBI:17015
cyclic-AMP	CHEBI:17489	Nicotinamide	CHEBI:17154
Deoxyadenosine	CHEBI:17256	Niacinamide	CHEBI:17155
dGMP	CHEBI:16192	NAD	CHEBI:13389
GMP	CHEBI:17345	NADH	CHEBI:16908
dGDP	CHEBI:28862	NADP	CHEBI:25524
7-methylguanosine	CHEBI:20794	Nicotinamide ribotide	CHEBI:50383
Xanthosine	CHEBI:18107	Methylnicotinamide	CHEBI:64399
Hypoxanthine	CHEBI:17368	Coenzyme A	CHEBI:15346
Purine	CHEBI:35584	Flavin adenine dinucelotide	CHEBI:24040
IMP	CHEBI:17202	Acetoacetate	CHEBI:13705
IDP	CHEBI:17808	Cholesteryl sulfate	CHEBI:41321
Inosione	CHEBI:17596	Glycerate	CHEBI:16659
Deoxyinosine	CHEBI:28997	sn-Glycerol-3-phosphate	CHEBI:15978
Cytidine	CHEBI:17562	Uric acid	CHEBI:27226
Cytosine	CHEBI:16040	Carbamoyl phosphate	CHEBI:17672
CMP	CHEBI:17361	Carbamaoyl aspartate	CHEBI:32814
CDP	CHEBI:17239	Maleic acid	CHEBI:18300
UMP	CHEBI:16695	Parahydroxybenzoate	
Orotate	CHEBI:30839	Acetylphosphate	CHEBI:13711
Dihydroorotate	CHEBI:30867	2-Keto-isovalerate	CHEBI:11851
Deoxyribose-phosphate	CHEBI:19569	Methylmalonic acid	CHEBI:30860
Uridine	CHEBI:16704	Pyroglutamic acid	CHEBI:16010
3-Amino isobutanoate	CHEBI:33094	Citraconic acid	CHEBI:17626
β-Hydroxybutyrate	CHEBI:8298	2-Ketohaxanoic acid	CHEBI:17308
N-Acetyl-L-alanine	CHEBI:40992	D-Gluconate	CHEBI:18391
Hydroxyisocaproic acid	CHEBI:55535	Glucose-1-phosphate	CHEBI:16077
p-Hydroxybenzoate	CHEBI:17879	N-Acetyl-glucosamine-1-phosphate	CHEBI:27625
Acetylphosphate	CHEBI:22191	UDP-D-glucose	CHEBI:18066
Phenylpropiolic acid	HMDB02359	UDP-D-glucuronate	CHEBI:17200
2-Hydroxy-2-methylbutanedioic acid	CHEBI:29003	UDP-N-acetyl-glucosamine	CHEBI:16264
Allantoin	CHEBI:15676	Citrate	CHEBI:35808
Indole-3-carboxylic acid	CHEBI:24809	Aconitate	CHEBI:16383
Phenylpyruvate	CHEBI:18005	Oxoglutarate	CHEBI:16810
Atrolactic acid	CHEBI:50392	Succinate	CHEBI:30779
Phenyllactic acid	CHEBI:25998	Fumarate	CHEBI:29806
Allantoate	CHEBI:17536	Malate	CHEBI:15595
2-Isopropylmalic acid	CHEBI:28635	Oxaloacetate	CHEBI:16452
Pyrophosphate	CHEBI:29888	2-Hydroxyglutarate	CHEBI:11596
Hydroxyphenylpyruvate	CHEBI:36242	Myo-inositol	CHEBI:17268
Indoleacrylic acid	HMDB00734	Pantothenate	CHEBI:16454
Xanthurenic acid	CHEBI:10072	Taurine	CHEBI:15891
dTDP	CHEBI:18075		
dGDP	CHEBI:28862		
d-glucose	CHEBI:17634		
Hexose-phosphate	CHEBI:47878		
Glucose-6-phosphate	CHEBI:17719		
Fructose-6-phosphate	CHEBI:78697		
Fructose-1,6-bisphosphate	CHEBI:78682		
D-Glyceraldehdye-3-phosphate	CHEBI:17138		
Dihydroxy-acetone-phosphatE	CHEBI:16108		
Phosphoglyceric acid	CHEBI:24346		
Phosphoenolpyruvate	CHEBI:18021		
Lactate	CHEBI:24996		
Glucono-D-lactone	CHEBI:24267		
6-Phospho-D-gluconate	CHEBI:16863		
Ribose-phosphate	CHEBI:26562		
Phosphoribosyl pyrophosphate	CHEBI:48956		
D-Erythrose-4-phosphate	CHEBI:48153		
Sedoheptulose 7 phosphate	CHEBI:15721		
D-Sedoheptulose-1-7-phosphate	CHEBI:17969		

### Reproducibility of Microscale Culture-Based Metabolomic Trends

**Fig 1:**
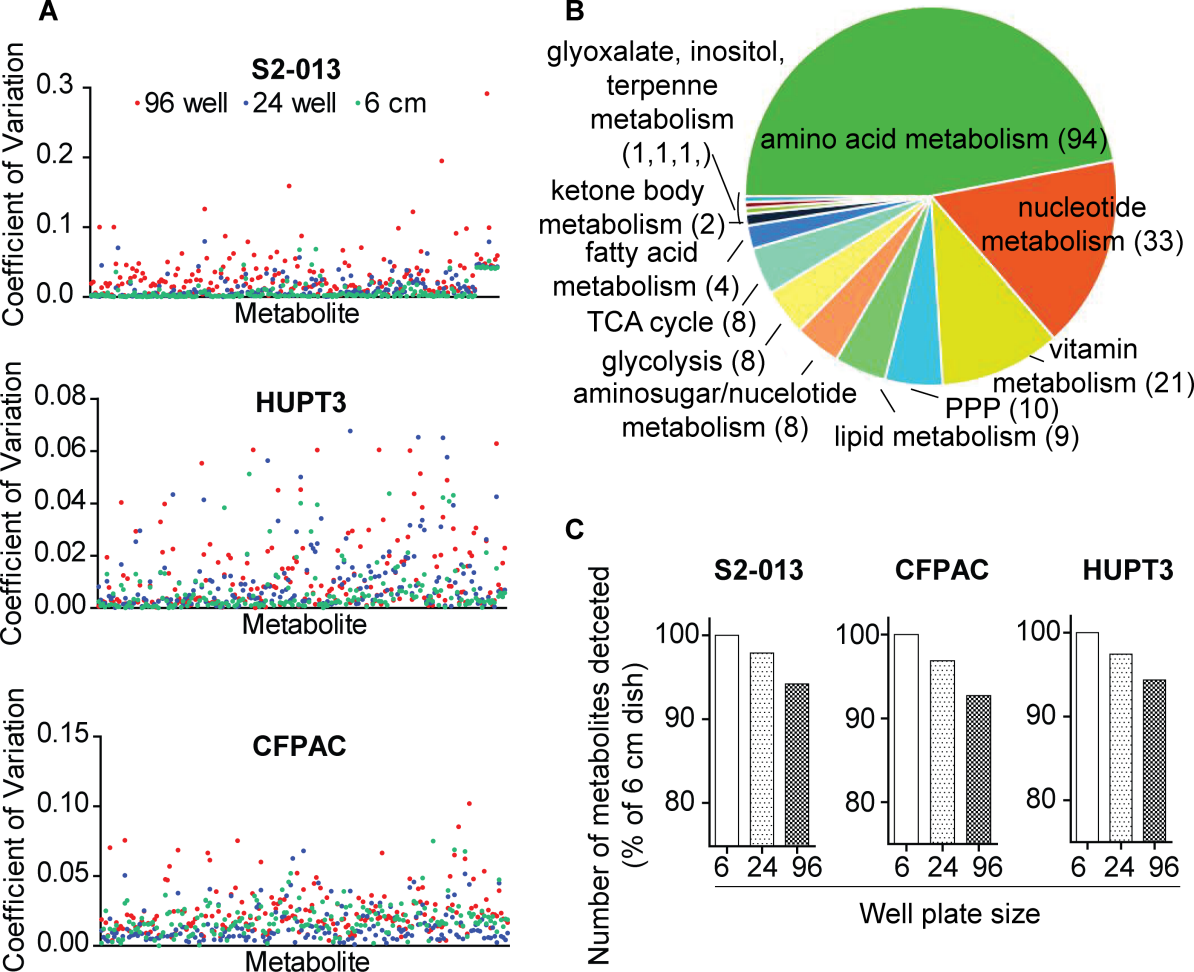
Reproducibility of metabolite detection from microscale cultures. (A) Scatter plots showing coefficient of variations for individual metabolites detected from 96-, 24-well and 6 cm culture dishes of 3 different pancreatic cancer cell lines. (B) Pie chart showing metabolite abundances corresponding to different metabolic pathways detected in-common from cell lysates. (C) Relative percentage of total number of metabolites detected from three different pancreatic cancer cell lines and three different-sized culture dishes are presented as bar graphs.

**Fig 2:**
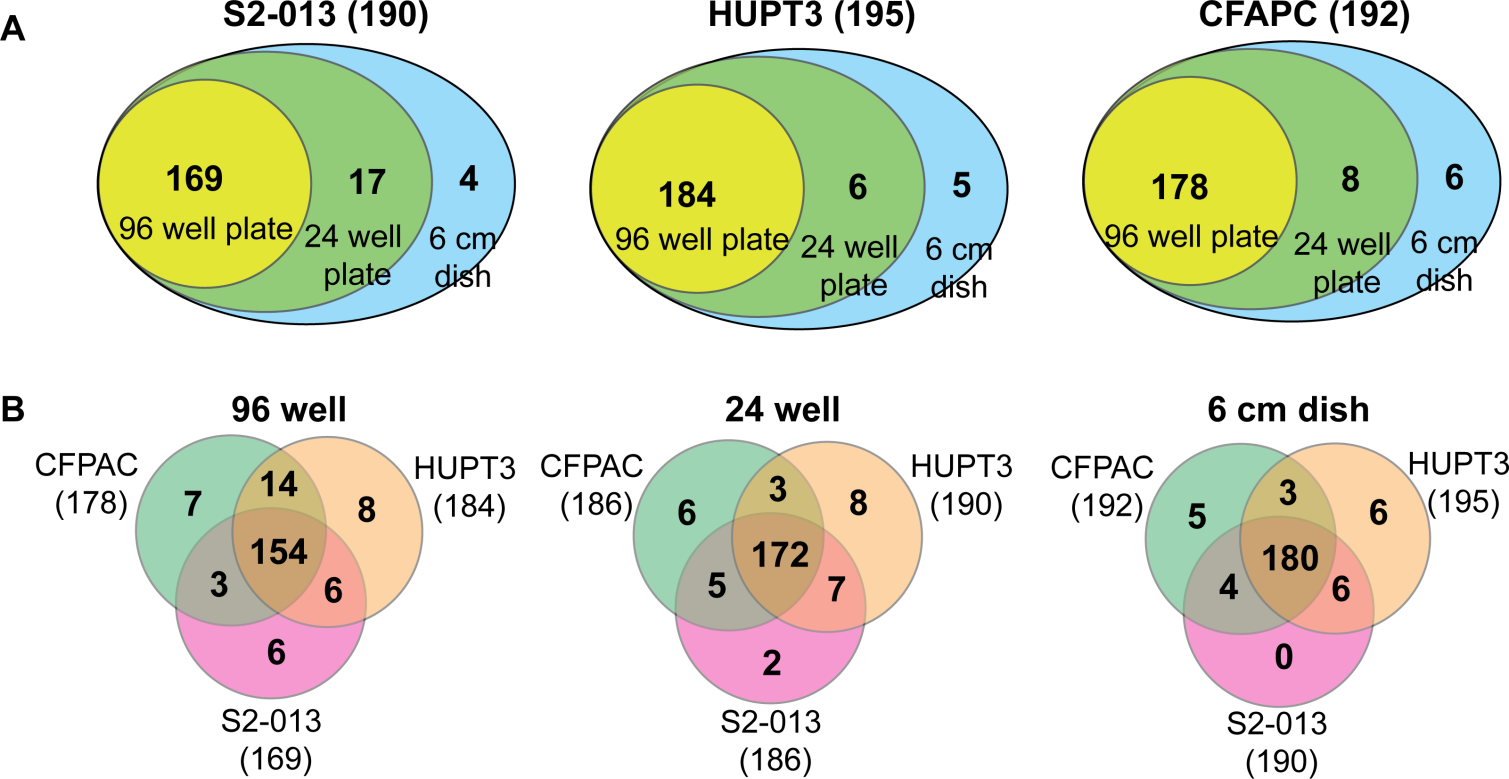
Cell type and decreased cell number modestly affect the total number of metabolites detected. (A) Venn diagrams representing number of common and unique metabolites detected in cell lysates obtained from each of the three different-sized culture dishes of individual cell lines. (B) Venn diagrams representing the number of common metabolites detected from cell lysates from each of the three different cell lines cultured in same-sized culture dishes. Numbers in parentheses indicate the total number of metabolites detected in the respective sample. The number of metabolites detected in each cell line and culture dishes are representative of individual replicates of each experiment.

The reproducibility of our metabolomics approach with microscale cultures was evaluated through the coefficient of variation (CV) analysis, which indicates variability among the metabolite peaks in metabolomic analyses [21]. Metabolite peak intensities obtained from 6 cm, as well as 24- and 96-well plate-culture dishes from three different pancreatic cancer cell lines demonstrated low CV values (Fig 1A, with a maximum CV < 0.3), indicating low variability among the replicates during metabolomic analyses. Metabolites detected with such low variability were inclusive of different metabolic pathways, with majority of the pathways encompassing amino acid metabolism followed by other metabolic pathways, as shown in Fig 1B and S1 Table, The frequency of detection of these metabolites from similar cell numbers using the three different pancreatic cancer cell lines was further analyzed to evaluate the reproducibility of the microscale metabolomic approach. Specifically, acquiring similar cell numbers from different cell lines was achieved by optimizing the experimental design. Metabolite extraction was performed from similar, pre-quantified cell numbers by applying proportionate volumes of extraction buffers to extract metabolites from the different sized culture plates. Comparative analysis of the total number of metabolites detected within each of the three cell lines indicated similar trends in metabolite abundancies from microscale cultures. The total number of detected metabolites decreased modestly with microscale cultures of all the three cell lines in the following order for extracts obtained from: 6 cm dishes > 24-well plates > 96-well plates, as shown in Fig 1C. Specifically, comparison of the percent of metabolites detected in cell lysates obtained from different cell numbers revealed the presence of 93 ± 1% of the metabolites from as few as 10,000 cells for the three pancreatic cancer cell lines examined. This percentage of metabolites slightly increased to 97% for 10^5^ cells from the three cell lines. The greatest number of metabolites was detected in the 10^6^ cells for HUPT3 cells. Notably, 96-well plate lysates of the same cell line also contained an increased number of detectable metabolites, as shown in Fig 2B. As such, we corroborated the reproducibility of our findings using microscale culture-based metabolomics with three different cell lines cultured under similar conditions, which showed similar trends and only a modest decrease in detectable metabolites corresponding to a decrease in cell numbers (Fig 1C).

### Effects of Cell Type on Metabolomic Trends

In order to avoid metabolomic perturbations that can arise due to cell types of different origins, or cell types of similar origins with different phenotypic or molecular characteristics [17,22], data analysis was confined to cell types of same cancer origin. Despite this confinement to cancer cell lines of a similar pathological origin, the frequency of metabolites detected varied with the cell number among the three cell lines (Fig 2A and 2B), which may be due to differential genetic and metabolic compositions in pancreatic cancer cell lines, as previously suggested [23,24]. Metabolites with signal intensities less than the set threshold (*i*.*e*., S/N < 10^4^) were predominantly found in the 96-well plates, followed by the 24-well plates (*i*.*e*., 10^5^ cells), which is possibly due to the reduced number of cells present in the 96-well plates (*i*.*e*., 10^4^). Interestingly, the frequency of common metabolites detected for equal cell numbers from the three different cell lines indicated that a maximum number of metabolites were common among similar numbers of cell lysates. Furthermore, the number of common metabolites detected from lysates of three cell lines was reduced in the 96-well plates (10,000 or 10^4^ cells) compared to 10^5^ and 10^6^ cells (Fig 2B). These findings indicate that the cell type and cell number both modestly affect the frequency of metabolite detection in metabolomic analysis.

### Different Effects of Dilution and Microscale Cultures on Metabolite Peak Intensities

**Fig 3:**
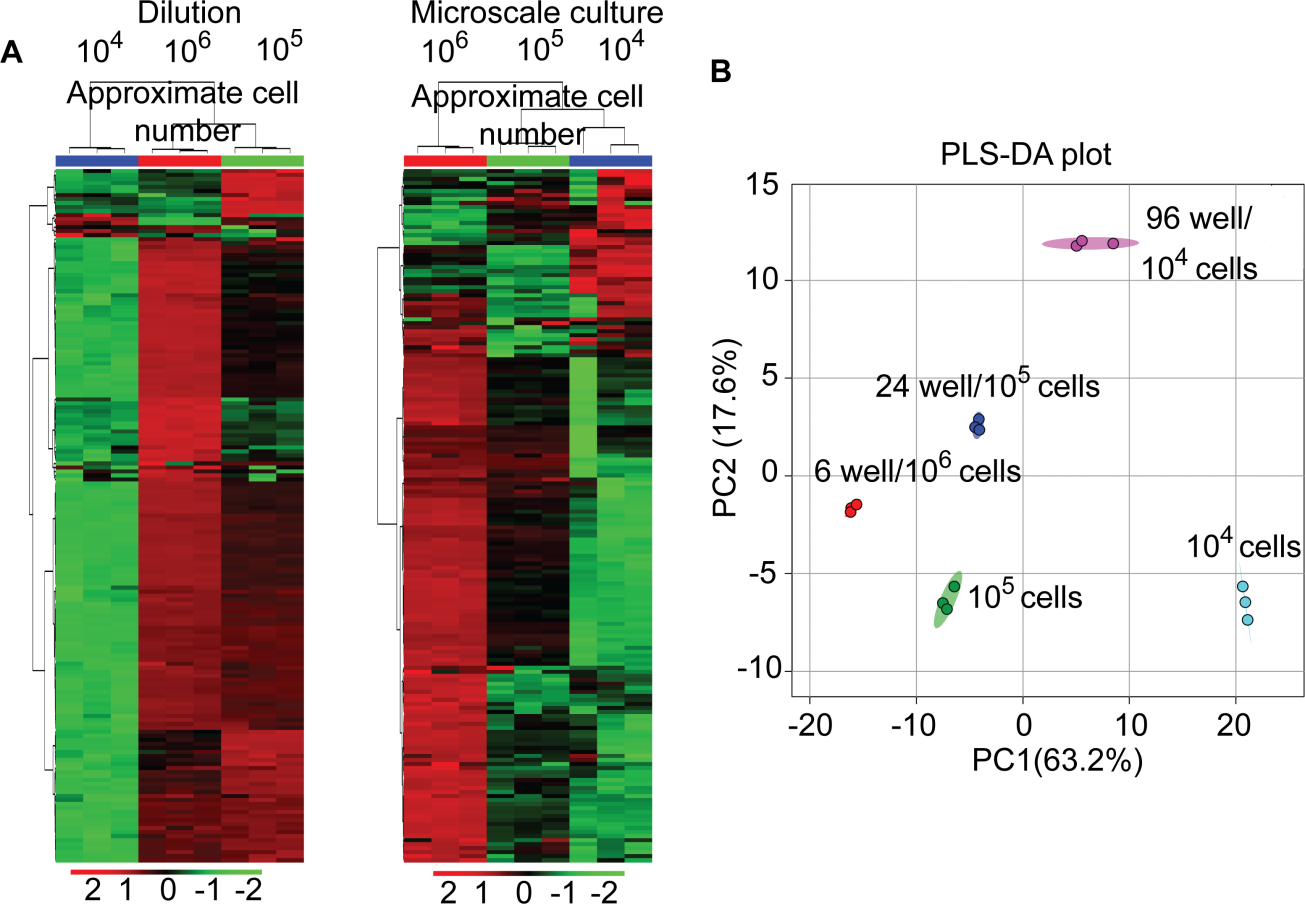
Differences between dilution and microscale culture metabolomic profiles. (A) Heat-maps showing unsupervised hierarchical clustering of diluted (left panel) and microscale culture (right panel) metabolite peak intensities. Each column in heat maps indicates a technical or biological replicate. Blue, red and green colored bar on the top of heat-maps indicate different groups used for analysis. (B) PLS-DA plot showing differential clustering of dilution (10^5^ and 10^4^ cells)and microscale-cultured samples (24 well and 96 well) based on total metabolite peak intensities. Individual clusters contain three technical or biological replicates as indicated by different color signs.

The effects of reduced cell numbers on metabolomic profiling has been reported using serially diluted samples [13]. Building on this study, we compared trends in peak intensities for decreasing cell numbers between diluted and microscale culture samples independently, using unsupervised hierarchical clustering analyses. Reproducibility of our LC-MS/MS methodology was demonstrated from the close clustering of the technical replicates obtained from the serially diluted samples and the biological replicates obtained through direct extraction of samples using unsupervised hierarchically clustered heat maps as shown in Fig 3A. Results from the unsupervised hierarchical clustering of replicates also indicated that both dilution and microscale methodologies result in descending trends for most metabolite peak intensities and corresponded to a decrease in cell numbers, for both serially diluted and directly extracted samples. As expected, metabolite abundances decreased primarily in the order of 10^6^ > 10^5^ > 10^4^ cells (Fig 3A), which was again in agreement with a previous report using serially diluted samples [13]. However, a few metabolites extracted from the reduced number of cells exhibited either an increase in peak intensities or no change. Notably, partial least squares discriminant analysis (PLS-DA) of metabolite groups showed segregation between the dilution and microscale culture extracts on the opposite axes of the second component (Fig 3B), indicating that differences were present in total metabolite peak intensities among the groups analyzed. Further, distinct segregation was observed based on both the cell number and the method of sample preparation, with the samples from same cell numbers but different plating densities segregating distinctly from one another on the PC2 axis and a minimum overlap on the PC1 axis (Fig 3B). This differential segregation of cell lysates from cells with equal numbers was reflecting the effect of sample preparation methods (direct extraction and dilution) on total metabolite profiles. Furthermore, the difference in metabolite profiles from 10^5^ cells obtained through two different methods of sample preparation was relatively lower than the differences between metabolite profiles from 10^4^ cell samples indicating that sample preparation method had a major impact on metabolite profiles from cells with lower numbers

## The Effect of Decreased Cell Numbers on the Linearity of Metabolite Abundance

**Fig 4:**
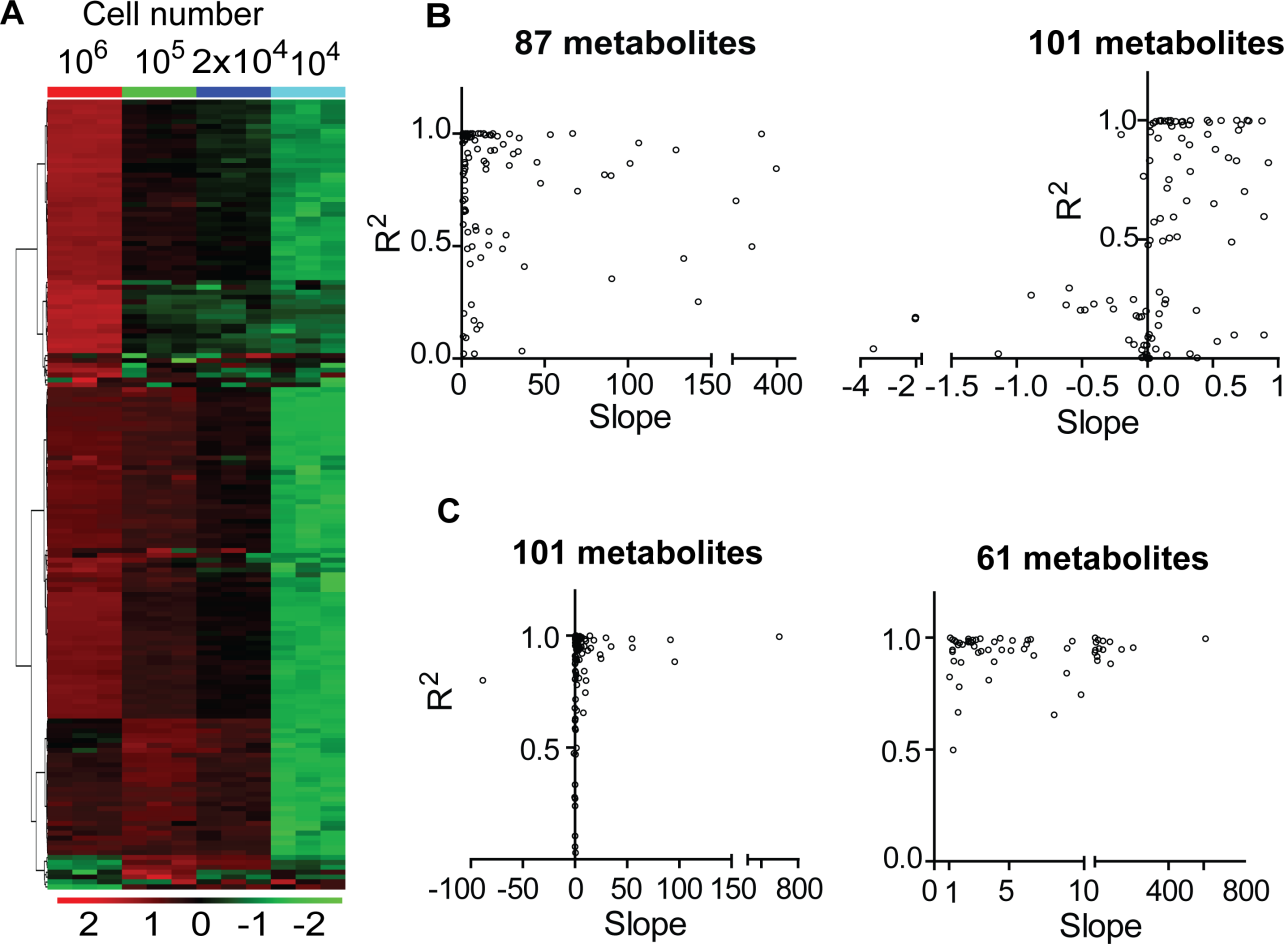
Effect of decreased cell numbers on metabolite peak intensities. (A) Heat-map showing unsupervised hierarchical clustering of metabolite peak intensities from differentially diluted samples. Each column in heat map indicates a biological replicate. Blue, cyan, red and green colored bars on the top of heat-map indicate different groups used for analysis. (B and C) Scatter plots showing correlation among slopes and corresponding coefficients of regression (R^2^) for individual metabolites that were obtained through non-linear regression analyses of metabolite peak intensities corresponding to the dilution of samples. Each dot in the scatter plots represents an individual metabolite. (B) Represents data corresponding to four different dilutions and cell numbers (10^6^, 10^5^, 2x10^4^ and 10^4^ cells) and (C) represents data corresponding to three different dilutions and cell numbers (10^5^, 2x10^4^ and 10^4^ cells), respectively.

**Fig 5:**
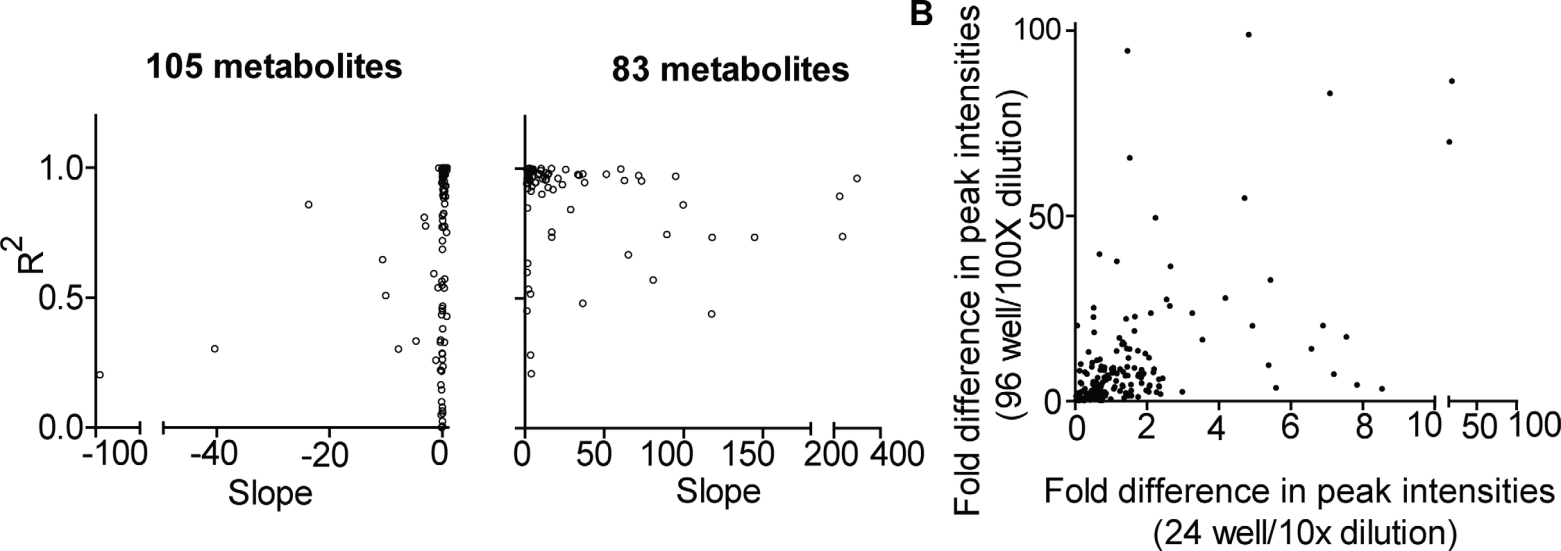
Microscale cultures alter metabolite intensities in metabolomic analyses. (A) Scatter plots showing correlation among slopes and corresponding coefficients of regression (R^2^) for individual metabolites that were obtained through non-linear regression analyses of metabolite peak intensities corresponding to the microscale-cultured samples after exclusion of increased cell number-peak intensity values. (B) Scatter plot showing correlations among fold differences in peak intensities of individual metabolites between different cell numbers. Each dot in the scatter plots represents an individual metabolite.

The initial analysis of peak intensities using serially diluted cell samples showed that some metabolite peak intensities did not decrease with sample dilution (Fig 3A, left panel). We further analyzed the linearity of metabolite abundances for the sample dilutions by comparing peak intensities obtained through four different dilutions. This comparison indicated that certain metabolite intensities either increased or were not altered by sample dilutions in S2-013 cell line (Fig 4A). More than 50% (*i*.*e*., 101 of 182) of the metabolites detected through sample dilutions exhibited either a minor increase in the slope or non-linear correlations based on the sample dilution (Fig 4B, left panel compared to right panel). The slopes of linear regression curves for 101 metabolites detected were either < 1 or negative, indicating that metabolite peak intensities did not increase corresponding to the concentration of the samples (Fig 4B, right panel). We further extended this analysis by excluding the peak intensities from higher cell numbers (*i*.*e*., 10^6^ cells in the 6-well plates) and found that linearity corresponded to an increase in cell numbers, with almost 99.5% of metabolites detected commonly among the diluted samples of S2-013 cell line (Fig 4C). In addition, excluding peak intensities obtained from samples with higher cell numbers (10^6^ cells) increased the fraction of metabolites showing positive slopes; the corresponding increase based on the cell number was by a factor of 10 (Fig 4C, see right panel compared to left panel). Given the similarities in the relative cell numbers of the diluted samples compared to the microscale cultures, we expected similarities in the patterns exhibited by metabolite abundances between these samples. As expected, among the total 188 metabolites detected common among the samples of microscale culture extracts, the analysis of linearity in metabolite peaks for the abundance of microscale culture extracts revealed that 105 metabolites had slopes less < 1, and 83 metabolite peaks had slopes of > 1, as seen in Fig 5A. Furthermore, using a relative fold analysis method, this study demonstrated that the majority metabolite peak intensities from similar numbered samples, obtained through direct extraction and dilution methods, were higher for the microscale culture extracts compared to peak intensities for the diluted samples (Fig 5B).

In general, metabolomic analyses utilize metabolite extracts obtained from cells that are cultured in 10 cm dishes [11,12]. The average cell count in a 10 cm dish ranges from 5–10 million, providing ample cell lysates that are re-suspended in 100–200 μl of solvents, and utilized for metabolomic analyses. However, a final sample volume of 2–5 μl of the re-suspended metabolite extract is actually injected into the LC-MS/MS system for metabolomic analysis. This sample volume falls within the general range of microliter to nanoliter samples required for metabolomic analyses [25]. Thus, the final volumes (2–5 μl) of samples required for metabolomic analyses represent a much smaller fraction of cells harvested from larger pools. This rationale supports the applicability of microscale cultured cell lysates as alternative sources for metabolomic analyses. While working with precious specimens or for analyzing the metabolomic changes due to a large series of treatments, microscale cultures could save a lot of resources and speed up the experiments. Despite their potential for metabolomic studies, microscale cultures are least utilized for omics-based metabolomic approaches in comparison to their application in metabolic evaluations for oxygen consumption and extracellular acidification experiments which are linked with cellular metabolism [26]. Our study addressed this gap in the applicability of microscale cultures for metabolomic platforms. We demonstrate that extracts corresponding to 10^4^ cells represent 93% of the metabolite peaks detected from higher cell numbers (10^6^). This microscale based metabolomic methodology can be extended to the metabolomic analyses preformed on samples with low cell numbers such as stem cells or immune cells isolated from clinical specimens, cells cultured in co-culture techniques and organoid cultures where low sample size and economic factors play critical role in metabolomic analyses.

*In vitro* cultures constitute standard culture platforms providing samples with the potential of genetic and therapeutic manipulations with great reproducibility for metabolomic studies. The standard ratio for the cell number-to-supplemented medium is provided through *in vitro* culture methods designed to yield samples that are optimal for metabolomic analyses. Although microscale cultures have the potential economic advantage over cultures with an increased cell density, it is important to note that cellular metabolomes in microscale cultures can be affected by the cell number-to-medium ratio. Taking this into account, our methodology consisted of culturing cells for 12 hours in 200, 500, and 5000 μl of media in 96-well, 24-well and 6 cm culture dishes, respectively (please see Methods section for more details). The cell number-to-medium ratios obtained using our approach correspond to 100 cells/μl for each well in 96-well plate and 400 cells/μl for each well in 24-well plate as well as for the 6 cm dish. The difference in peak intensities observed among similar numbered cell samples (Fig 3) is potentially due to different cell number-to-media ratios obtained in our method. Among the serial dilution samples, dilutions at 10x and 100x corresponded to a proportionate decrease in cell number-to-media ratios, which theoretically would correspond to 40 cells/μl and 10 cells/μl, respectively. Surprisingly, these ratios increased in microscale cultures for similar cell numbers (*i*.*e*., ratios of 4:400 for 10^5^ cells in 10x dilution vs. 24-well cultures, and 10:100 for 10^4^ cells in 100x dilution compared to 96-well cultures). These ratios have the potential to increase peak intensities for microscale cultures, in addition to the accumulation of metabolites in microscale culture samples. Thus, the differences in peak intensities for the same metabolites among the similar number of cells from samples obtained through different methods contradicts the assumption that the metabolome of cultures at the microscale reflects the serial dilution of samples, as previously shown [13]. In summary, our analyses indicate that biological factors, such as cell type and the ratio of cell number-to-media, play a critical role in metabolomic analysis performed utilizing cell lines cultured *in vitro*.

## Conclusions

The study presented herein examined effects that reduced numbers of cells have on targeted, quantitative metabolite profiling for reduced cell numbers. We performed direct comparison of metabolomic profiles obtained through the dilution and direct microscale extraction for reduced cell numbers. Based on an extensive review of the literature, this is the first study to demonstrate that metabolite profiles of microscale cultures cannot be extrapolated to those of high cell density extracts, which implies that dilution sample metabolite profiles cannot mirror microscale culture metabolite profiles. These differences are possibly due to the differences in the ratios of cell number-to-medium that can arise in such experimental procedures. On the other hand, this study corroborates the utility of microscale culture methods in metabolomics, particularly considering that the total number of detectable metabolites is only modestly affected by the decrease in cell numbers. Thus, our findings support the utilization of microscale cultures for metabolomics applications, opening a portal for new paradigms to be economically explored based on microscale culture-based metabolomics.

## Supporting Information

S1 Table Summary of metabolites and corresponding pathways (DOCX)
