# Supplementary material for: Validation of Metabolic Alterations in Microscale Cell Culture Lysates Using Hydrophilic Interaction Liquid Chromatography (HILIC)-Tandem Mass Spectrometry-Based Metabolomics
Source: PLoS One. 2016 Apr 27;11(4):e0154416. doi: 10.1371/journal.pone.0154416 (PMC4847783; doi:10.1371/journal.pone.0154416)
Supplement: S1 Table — (DOCX) [file pone.0154416.s001.docx]

**Supplementary Information**

**S1 Table: Summary of metabolites and corresponding pathways**

| **Metabolite** | **Metabolic pathway** | **Metabolite** | **Metabolic pathway** |
| --- | --- | --- | --- |
| Isobutyrylglycine | Amino acid metabolism | creatine | amino acid metabolism |
| Serine | Amino acid metabolism | creatinine | amino acid metabolism |
| 3-Phospho-serine | Amino acid metabolism | arginine | amino acid metabolism |
| Phosphoserine | Amino acid metabolism | 1,4-diaminobutane | amino acid metabolism |
| Betaine | Amino acid metabolism | ornithine | amino acid metabolism |
| Betaine aldehyde | Amino acid metabolism | citrulline | amino acid metabolism |
| Dimethylglycine | Amino acid metabolism | allaontoin | amino acid metabolism |
| Threonine | Amino acid metabolism | Urea | amino acid metabolism |
| Lysine | Amino acid metabolism | sarcosine | amino acid metabolism |
| Pipecolic acid | Amino acid metabolism | L-arginino-succinate | amino acid metabolism |
| Carnitine | Amino acid metabolism | N-Acetylputrescine | amino acid metabolism |
| Acetylcarnitine | Amino acid metabolism | carnosine | amino acid metabolism |
| L-alpha-Aminoadipate | Amino acid metabolism | N-acetyl-L-ornithine | amino acid metabolism |
| Acetyllysine | Amino acid metabolism | histidine | amino acid metabolism |
| Alanine | Amino acid metabolism | 3 methyl histidine | amino acid metabolism |
| Asparagine | Amino acid metabolism | 1-Methylhistamine | amino acid metabolism |
| Aspartate | Amino acid metabolism | Imidazoleacetic acid | amino acid metabolism |
| Phenylalanine | Amino acid metabolism | 1-Methyl-Histidine | amino acid metabolism |
| Tyrosine | Amino acid metabolism | Proline | amino acid metabolism |
| Dopamine | Amino acid metabolism | hydroxyproline | amino acid metabolism |
| Valine | Amino acid metabolism | glutamine | amino acid metabolism |
| Methylhydroxyisobutyrate | Amino acid metabolism | glutamate | amino acid metabolism |
| Leucine | Amino acid metabolism | Phenylacetylglutamine | amino acid metabolism |
| Lipoamide | Amino acid metabolism | tryptophan | amino acid metabolism |
| Methylsuccinic acid | Amino acid metabolism | kynurenine | amino acid metabolism |
| Methionine | Amino acid metabolism | 4-aminobutyrate | amino acid metabolism |
| Methionine sulfoxide | Amino acid metabolism | Indole | amino acid metabolism |
| Dimethyl-L-arginine | Amino acid metabolism | beta amino butyric acid | amino acid metabolism |
| Homocysteine | Amino acid metabolism | Melatonin | amino acid metabolism |
| Cystathionine | Amino acid metabolism | Metanephrine | amino acid metabolism |
| Cysteamine | Amino acid metabolism | gamma-Aminoisobutyrate | amino acid metabolism |
| Methylcysteine | Amino acid metabolism | Tryptophanol | amino acid metabolism |
| S-adenosyl-L-homocysteine | Amino acid metabolism | 3-OH-anthranilate | amino acid metabolism |
| S-adenosyl-L-methionine | Amino acid metabolism | hydroxy-Tryptophan | amino acid metabolism |
| S-adenosyl-L-methioninamine | Amino acid metabolism | 3-amino isobutanoate | amino acid metabolism |
| Glutathione | Amino acid metabolism | 2-Aminooctanoic acid | amino acid metabolism |
| Glutathione disulfide | Amino acid metabolism | L-alpha-Aminobutyrate | amino acid metabolism |
| S-ribosyl-L-homocysteine | Amino acid metabolism | Pyroglutamic acid | amino acid metabolism |
| Citraconic acid | Amino acid metabolism | fructose-1,6-bisphosphate | glycolysis\PPP |
| 2-ketohaxanoic acid | Amino acid metabolism | D-glyceraldehdye-3-phosphate | glycolysis\PPP |
| N-Acetyl-L-alanine | Amino acid metabolism | mesaconic acid | Glyoxylate and dicarboxylate metabolism |
| Hydroxyisocaproic acid | Amino acid metabolism | myo-inositol | inositol metabolism |
| P-hydroxybenzoate | Amino acid metabolism | beta hydroxybutyrate | ketone body metabolism |
| Acetylphosphate | Amino acid metabolism | acetoacetate | ketone body metabolism |
| Phenylpropiolic acid | Amino acid metabolism | Phosphorylcholine | lipid metabolism |
| 2-Hydroxy-2-methylbutanedioic acid | Amino acid metabolism | Choline | lipid metabolism |
| Allantoin | Amino acid metabolism | Glycerophosphocholine | lipid metabolism |
| Indole-3-carboxylic acid | Amino acid metabolism | coenzyme A | lipid metabolism |
| Phenylpyruvate | Amino acid metabolism | cholesteryl sulfate | lipid metabolism |
| Atrolactic acid | Amino acid metabolism | glycerate | lipid metabolism |
| Phenyllactic acid | Amino acid metabolism | sn-glycerol-3-phosphate | lipid metabolism |
| Allantoate | Amino acid metabolism | parahydroxybenzoate | lipid metabolism |
| 2-Isopropylmalic acid | Amino acid metabolism | aminoimidazole carboxamide ribonucleotide | nucleotide metabolism |
| Hydroxyphenylpyruvate | Amino acid metabolism | adenosine | nucleotide metabolism |
| Indoleacrylic acid | Amino acid metabolism | 1-Methyladenosine | nucleotide metabolism |
| Xanthurenic acid | Amino acid metabolism | S-methyl-5-thioadenosine | nucleotide metabolism |
| D-gluconate | Amino asugar and nucelotide metabolism | adenine | nucleotide metabolism |
| Glucose-1-phosphate | Amino asugar and nucelotide metabolism | AMP | nucleotide metabolism |
| N-acetyl-glucosamine-1-phosphate | Amino asugar and nucelotide metabolism | dAMP | nucleotide metabolism |
| UDP-D-glucose | Amino asugar and nucelotide metabolism | deoxyadenosine | nucleotide metabolism |
| UDP-D-glucuronate | Amino asugar and nucelotide metabolism | dGMP | nucleotide metabolism |
| UDP-N-acetyl-glucosamine | Amino asugar and nucelotide metabolism | GMP | nucleotide metabolism |
| Glucosamine | Amino sugar and nucleotide sugar metabolism | 7-methylguanosine | nucleotide metabolism |
| N-acetyl-glucosamine | Amino sugar and nucleotide sugar metabolism | xanthosine | nucleotide metabolism |
| Maleic acid | Fatty acid metabolism | hypoxanthine | nucleotide metabolism |
| Acetylphospahte | Fatty acid metabolism | Purine | nucleotide metabolism |
| 2-keto-isovalerate | Fatty acid metabolism | IMP | nucleotide metabolism |
| Methylmalonic acid | Fatty acid metabolism | inosione | nucleotide metabolism |
| D-Glucose | Glycolysis | deoxyinosine | nucleotide metabolism |
| Hexose-phosphate | Glycolysis | cytidine | nucleotide metabolism |
| Glucose-6-phosphate | Glycolysis | cytosine | nucleotide metabolism |
| PGA | Glycolysis | CMP | nucleotide metabolism |
| Phosphoenolpyruvate | Glycolysis | NAD | vitamin metabolism |
| Lactate | Glycolysis | NADH | vitamin metabolism |
| Dihydroxy-acetone-phosphate | Glycolysis\lipid metabolism | UMP | nucleotide metabolism |
| F6P | Glycolysis\PPP | Uric acid | nucleotide metabolism |
| Dihydroorotate | Nucleotide metabolism | Orotate | nucleotide metabolism |
| Deoxyribose-phosphate | Nucleotide metabolism | orotidine-5-phosphate | nucleotide metabolism |
| Uridine | Nucleotide metabolism | NADP | vitamin metabolism |
| DTDP | Nucleotide metabolism | Nicotinamide ribotide | vitamin metabolism |
| DGDP | Nucleotide metabolism | methylnicotinamide | vitamin metabolism |
| CDP | Nucleotide metabolism | flavin adenine dinucelotide | vitamin metabolism |
| ADP | Nucleotide metabolism | Carbamoyl phosphate | vitamin metabolism |
| IDP | Nucleotide metabolism | carbamaoyl asparatate | vitamin metabolism |
| Cyclic-AMP | Nucleotide metabolism | pantothenate | vitamin metabolism |
| Glucono-D-lactone | PPP | Taurine | vitamin metabolism |
| 6-phospho-D-gluconate | PPP | thiamine | vitamin metabolism |
| Ribose-phosphate | PPP | riboflavin | vitamin metabolism |
| Phosphoribosyl pyrophosphate | PPP | 5 methyl THF | vitamin metabolism |
| D-Erythrose-4-phosphate | PPP | nicotinamide | vitamin metabolism |
| Sedoheptulose 7 phosphate | PPP | Niacinamide | vitamin metabolism |
| D-Sedoheptulose-1-7-phosphate | PPP |  |  |
| Citrate | TCA cycle |  |  |
| Aconitate | TCA cycle |  |  |
| Oxoglutarate | TCA cycle |  |  |
| Succinate | TCA cycle |  |  |
| Fumarate | TCA cycle |  |  |
| Malate | TCA cycle |  |  |
| Oxaloacetate | TCA cycle |  |  |
| 2-hydroxygluterate | TCA cycle |  |  |
| Mevalonic acid | Terpenoid metbaolism |  |  |
| Cobalamin | Vitamin metabolism |  |  |
| Pyridoxine | Vitamin metabolism |  |  |
| Pyridoxamine | Vitamin metabolism |  |  |
| Flavone | Vitamin metabolism |  |  |
| Biotin | Vitamin metabolism |  |  |
| Thiamine phosphate | Vitamin metabolism |  |  |
